# Recurrent pregnancy loss is associated to leaky gut: a novel pathogenic model of endometrium inflammation?

**DOI:** 10.1186/s12967-018-1482-y

**Published:** 2018-04-17

**Authors:** C. Tersigni, S. D’Ippolito, F. Di Nicuolo, R. Marana, V. Valenza, V. Masciullo, F. Scaldaferri, F. Malatacca, C. de Waure, A. Gasbarrini, G. Scambia, N. Di Simone

**Affiliations:** 1 Department of Woman and Child Health, A. Gemelli Hospital, Università Cattolica Del Sacro Cuore of Rome, 00168 Rome, Italy; 2 Department of Nuclear Medicine, A. Gemelli Hospital, Università Cattolica Del Sacro Cuore, A. Gemelli Hospital, 00168 Rome, Italy; 3 Department of Internal Medicine, A. Gemelli Hospital, Università Cattolica Del Sacro Cuore, 00168 Rome, Italy; 4 International Scientific Institute Paolo VI, ISI, A. Gemelli Hospital, Università Cattolica Del Sacro Cuore, 00168 Rome, Italy; 5 Institute of Public Health, A. Gemelli Hospital, Università Cattolica Del Sacro Cuore, 00168 Rome, Italy

**Keywords:** Recurrent pregnancy loss, Intestinal permeability, inflammasome, Human endometrium

## Abstract

**Background:**

Recurrent pregnancy loss (RPL) occurs in 3–5% in about 30% of cases no cause can be found. Women with RPL show higher prevalence of undiagnosed gut disorders. Furthermore, in endometrial tissues of RPL women, higher expression of pro-inflammatory cytokines and Nalp-3 inflammasome has been observed. Aim of this study was to investigate whether an abnormal gut permeability might occur in RPL women and allow passage into systemic circulation of pro-inflammatory molecules able to induce endometrial inflammation.

**Methods:**

70 women with idiopathic RPL and 30 healthy women were recruited at the Recurrent Pregnancy Loss Outpatient Unit of the Gemelli Hospital of Rome from March 2013 to February 2017. Enrolled women underwent ^51^Cr-ethylene-diamine-tetraacetic acid absorption test to evaluate intestinal permeability. Sera obtained from enrolled women were analysed for lipopolysaccharide (LPS) by ELISA. Anxiety and depression state were evaluated by administering STAI-Y and Zung-SDS tests, respectively. Of all recruited individuals, 35 women with idiopathic RPL and 20 healthy controls accepted to undergo diagnostic hysteroscopy and endometrial biopsy. Endometrial lysates were investigated for inflammasome Nalp-3 by Western blot analysis, and caspase-1, IL-1β and IL-18 by ELISA, respectively.

**Results:**

Higher prevalence of abnormal intestinal permeability (P < 0.0001), increased circulating levels of LPS (P < 0.05), anxiety (P < 0.05) and depression (P < 0.05) were observed in RLP women compared to controls. Endometrial expression of Nalp-3, caspase-1 and IL-1β was significantly increased in RPL group (P < 0.0001; P < 0.05 and P < 0.001, respectively). IL-18 endometrial levels were not found to be higher in RPL cases. Statistically significant association between higher intestinal permeability and abnormally increased expression of endometrial Nalp-3, was observed in RPL (P < 0.01). Furthermore, higher LPS serum levels, a bacterial-derived activator of Nalp-3 complex, was shown to be statistically associated to abnormal endometrial expression of Nalp-3 inflammasome (P < 0.01) in RPL women.

**Conclusions:**

In women with RLP, leaky gut might occur and allow passage into circulation of immune triggers, potentially able to elicit endometrial innate immune response and, thus, to contribute to miscarriage pathogenesis. Diagnosis and treatment of intestinal disorders underlying leaky gut might improve endometrial environment and pregnancy outcome.

## Background

Spontaneous pregnancy loss is a surprisingly common occurrence. Approximately 15–20% of all clinically recognized pregnancies result in spontaneous loss and it is believed that there are many more pregnancies, up to 40–50%, that fail prior to being clinically recognized [1]. For a single isolated miscarriage within the 10th week of gestation no tests should be proposed to couples. A completely different clinical condition is represented by recurrent pregnancy loss (RPL), which occurs in 3–5% of all women and is defined by three consecutive pregnancy losses prior to 23 weeks of gestational age [1].

Using evidence-based tests for evaluation of couples with RPL, approximately 60% of couples will have an etiology identified that could be associated with their losses. There are other factors, such as lifestyle factors, that may contribute to miscarriage and can be corrected or eliminated. These include maternal age, obesity, tobacco use and alcohol consumption. However, in about 40% of all RPL cases, after a complete screening for the known causes or predisposing factors, none of the above anomalies can be found, identifying cases of idiopathic RPL [1]. Then, despite a thorough evaluation for RPL, not all couples will have answers to their dilemma.

It is known that RPL has a significant emotional impact on couples: early pregnancy loss is usually a shocking and traumatic event for women and their families. During the initial weeks following a loss, symptoms of grief may be impossible to distinguish from depression, and some women may continue to experience depressive symptoms for months [2]. Contributing to the distress experienced after miscarriage is the fact that society may not recognize the significance of the loss to the parents. Recently, Kolte and coauthors found that both psychological stress and major depression are significantly more common among women with RPL than in those with regular pregnancies [3]. From a pathogenic point of view, it is worth of note that there are evidences showing that depression and inflammation are intertwined and feeding off each other. This bidirectional loop, in which depression facilitates inflammatory responses and inflammation promotes depression may have consequences on health. Indeed, elevated inflammatory signaling disregulates neurotransmitter metabolism, impairs neuronal health, and alters neural activity in mood-relevant brain regions [4, 5]. In particular, it has been shown that pro-inflammatory cytokines and lipopolysaccharide (LPS) may induce depressive symptoms [6–8]. On the other hand, depression is accompanied by an activation of the inflammatory response system. There is now evidence that intestinal mucosal dysfunction, characterized by an increased translocation of Gram-negative bacteria, called “leaky gut”, plays a role in the inflammatory pathophysiology of depression [9]. Furthermore, it is suggested that the increased LPS translocation, related to leaky gut, may induce an immune response and a systemic tissue inflammation in patients with depression and specific sickness behaviour symptoms [10].

Concerning pregnancy loss, the endometrial expression of pro-inflammatory cytokines (IL-1β, TNF-α, IFN-γ, and TGF-β1) has been shown to be up-regulated in women with idiopathic RPL compared with controls [11]. Moreover, recently, we observed an abnormal activation of the endometrial inflammasome Nalp-3, which represents the first line of defense against cellular stress and is a crucial component of innate immunity [12]. Upon activation of Nalp-3, apoptosis-associated speck-like protein containing a CARD (ASC) and caspase-1 are assembled and this multiprotein complex enables the caspase-1-mediated proteolytic processing of the pro-inflammatory cytokines (IL-1β and IL-18), generating their respective mature secretory forms. These events are necessary for the induction of further systemic responses and spreading of inflammation [13, 14].

Currently, there are no available data in the international literature on the role of abnormal intestinal permeability and RPL. Thus, since the clinical conditions characterized by increased intestinal permeability, also called “leaky gut”, may be related to the passage of pathogens-derived proteins (like LPS) into the blood circulation, therefore inducing inflammasome activation, the aims of this study were:

- to assess intestinal permeability and circulating LPS levels, as well as depression and anxiety levels, in women with RPL;
- to investigate endometrial expression and activation of Nalp-3 in the same patients, correlating tissue inflammation to abnormality of intestinal permeability and circulating levels of LPS.

## Methods

### Patients and samples

This case–control study was performed between March 2013 and February 2017 at the Recurrent Pregnancy Loss Outpatient Clinic of the Gemelli Hospital of Rome, Italy. The study population included 70 women with history of idiopathic RPL with three or more spontaneous consecutive pregnancy losses (≤ 12 weeks of gestation) clinically documented by ultrasonography [1] at least 6 months before the study and 30 healthy women with two or more previous uncomplicated term pregnancies (control group).

The inclusion criteria for both groups were as follows: caucasian, age ≤ 39 years, healthy, regular ovulatory cycles (28–32 days), normal serum levels of follicle-stimulating hormone (FSH < 10 mIU/ml), luteinizing hormone (LH < 10 mIU/ml) and anti-mullerian hormone (AMH > 2 ng/ml) on day 3 of the menstrual cycle, absence of abnormal ovarian and endometrial ultrasonographic features, no use of any contraceptive drugs or intrauterine device in the last 6 months. Exclusion criteria were: smoking, alcohol consumption, presence of abnormalities at the screening for RPL (anatomical abnormalities, endometrial cavity anomalies assessed by hysteroscopy, luteal phase deficiency, hyperprolactinemia, hyperinsulinemia or insulin resistance, endocrine disorders, vaginal infections, karyotype anomalies, thrombophilic disorders, autoimmune diseases), obesity, endometriosis, refusal to undergo intestinal permeability test, any pathological conditions potentially interfering with intestinal permeability, like celiac disease or gluten sensitivity, history of viral hepatitis, diarrhea, diverticulosis, irritable bowel syndrome, inflammatory bowel diseases, or bariatric or other abdominal surgery, immunoglobulin A or immunoglobulins deficiency, impaired renal function, use of non-steroidal anti-inflammatory drugs, antibiotics, probiotics, or anti-secretory drugs within the 3 months preceding enrollment.

### Samples collection

Peripheral blood samples (5 ml) from all RPL (n = 70) and healthy control women (n = 30) enrolled were collected by vein puncture. Samples were spun at 3000 g at 4 °C for 20 min, then the supernatant were aliquoted and stored at − 80 °C until use.

Thirty-five of seventy RPL women and twenty of thirty controls accepted a diagnostic mini-hysteroscopy during the putative window of implantation (days 19th to 24th), with endometrial biopsies. The biopsies timing was chosen according to the last menstrual period, monitoring the follicle size by transvaginal ultrasound and then confirmed by histologic assessment [15]. All women were advised to avoid sexual intercourses from menses to planned hysteroscopic procedure. Serum progesterone and β-hCG levels were determined just before hysteroscopy. Endometrial biopsy were performed using a 3-mm Novak curette, avoiding any contact between the curette and the vaginal walls, and then immediately washed in normal saline solution and stored at − 80 °C.

Biopsies were also analysed for *Chlamydia trachomatis*, *Mycoplasma*, *Ureaplasma urealyticum*, *Neisseria gonorrheae* or yeast colonization. Patients with endometrial samples positive for pathogens colonization as well as those with evidence of chronic endometritis at hysteroscopy were not included in the study.

### Anxiety and depression state evaluation

Acute or stable anxiety of enrolled women was assessed by administration of the State-Trait Anxiety Inventory (STAI) Y test [16, 17], divided in two parts: the first evaluating the anxiety state by inquiring current emotional state asking twenty questions with responses ranging from ‘‘not at all’’ (score 1) to ‘‘extremely’’ (score 4) on a 1–4 Likert scale. Total scores ranged from 20 to 80, with higher scores indicating higher degrees of state anxiety. The second part of STAI Y test assesses trait anxiety by asking to subjects to describe how they usually feel. Twenty questions were asked to enrolled patients with responses ranging from ‘‘almost never’’ (score 1) to ‘‘almost always’’ (score 4) on a Likert 1–4 scale. Higher scores reflected greater symptomatology and a suggested cutoff of 39 was established for clinically significant anxiety (score: 40–50 mild; 50–60 moderate; > 60 severe [17]. STAI -Y questionnaires were completed in approximately 20 min.

Depressive symptomatology was investigated with the use of the Zung Self-Rating Depression Scale (Z-SDS) [18], a short self-administered survey. The Z-SDS scale consists of 20 items rating the four common characteristics of depression: (1) the pervasive effect, (2) the physiological equivalents, (3) other disturbances and (4) psychomotor activities. Each question is scored on a scale of 1–4 [corresponding to (1) a little of the time, (2) some of the time, (3) good part of the time and (4) most of the time]. The scores range from 25 to 100 to be set on a depression scale with higher scores reflecting greater symptomatology: 25–49 Normal Range; 50–59 Mildly Depressed; 60–69 Moderately Depressed; 70 and above Severely Depressed.

## ^51^Cr-EDTA intestinal permeability test

^51^Cr-ethylene-diamine-tetraacetic acid (^51^Cr-EDTA) absorption test was performed in all patients enrolled to assess intestinal permeability as described elsewhere [19]. Enrolled women were all advised to avoid pregnancy during the test month. After an overnight fast, patients were administered 0.37 MBq ^51^Cr-EDTA (Amersham Health, England, UK) in 10 ml water. The radiation dose administered was very low, analogue to the dose coming from a 2 days exposure to natural background radiation. The 24-h urinary excretion of ^51^Cr-EDTA was analyzed using a γ-counter (LKB-Wallac 1282 Compugamma, Turku, Finland). Cr-EDTA clearance was calculated using the formula:

$$[({\text{mean urinary counts}} \times {\text{urinary volume}}) \times ({\text{standard counts}} \times 50) - 1].$$

Results were expressed as fraction of the oral administered dose and considered abnormal when ≥ 3% [19]. All women with abnormal intestinal permeability test (≥ 3%) underwent a gastroenterological evaluation and were then evaluated for serum levels of immunoglobulin (Ig) A and E, anti-endomysial, anti-gliadin, anti-transglutaminase, perinuclear and cytoplasmic anti-neutrophil cytoplasmic antibodies (pANCA, and cANCA, respectively), Anti-Smooth Muscle and Anti–Saccharomyces cerevisiae antibodies (ASMA and ASCA, respectively) antibodies, HLA DQ2-DQ8 haplotype and underwent lactose/lactulose/sorbitol breath test and fecal calprotectin assay to exclude all known causes of increased intestinal permeability.

### Assessment of circulating lipopolysaccharide levels

Sera from RPL and control women were thaw and analyzed for concentration of the Gram negative bacterial membrane component, LPS by LAL chromogenic endpoint assay (HyCult Biotechnology, Uden, Netherlands) according to the manufacturer’s instructions. Results were analysed by spectrophotometer (Titertek Multiscan plus Mk II plate reader ICN Flow Laboratories, Irvine, CA) measuring the absorbance at wavelengths of 450 nm.

### Western blot analysis of endometrial Nalp-3 expression

To investigate inflammasome Nalp-3 endometrial expression by Western blot, total cellular lysates obtained from endometrial biopsies of ten RPL and ten control women were separated by 10% SDS-PAGE electrophoresis under reducing conditions. After gel electrophoresis and transfer of proteins to a nitrocellulose membrane, nitrocellulose sheets were blocked at room temperature for 1 h in 5% non-fat dry milk and incubated overnight at 4 °C with a specific primary mouse monoclonal anti-Nalp-3 antibody (ThermoFisher Scientific, Rockford, IL, USA). The membranes were then washed with PBST and incubated with specific horseradish peroxidase-conjugated IgG diluted 1:2000 in PBST with 5% of non-fat dried milk. Bound secondary antibody was detected by chemiluminescence. Bands were analyzed by a Gel Doc 200 Image Analysis System and quantified with the Quantity One Quantitation Software (both from BioRad). As positive load control cytosolic β-actin band (42-kDa) was detected by a mouse monoclonal anti-human β-actin antibody (Sigma-Aldrich, St Louis, MO, USA).

### Endometrial assessment of caspase-1, IL-1β and IL-18

Caspase-1, IL-18 and IL-1β levels were measured in endometrial lysates from 35 RLP and twenty control women by a commercial enzyme-linked immunoassay (ELISA), according to manufacturer’s instructions (USCN Life Science Inc. and Cloud-Clone Corp. Houston, TX, USA). Briefly, 100 µl of samples or standard were added to each well coated with human monoclonal anti-caspase-1 or IL-18 or IL-1β antibodies. After 2 h of incubation at 37 °C, wells were washed and incubated with a specific enzyme-linked polyclonal antibody, conjugated to horseradish peroxidase. Then, tetramethyl-benzidine substrate solution was added to each well, and the color developed in proportion to the amount of the proteins bound in the initial step. The plate was read on a Titertek Multiscan plus Mk II plate reader (ICN Flow Laboratories, Irvine, CA) measuring the absorbance at wavelengths of 450 nm. Results were normalized for protein content of each sample.

### Statistical analysis

Results are expressed as mean ± standard deviation (SD). Data were analyzed using one-way analysis of variance (ANOVA) followed by a post–hoc test (Bonferroni test) or Chi square according to type of variables. Statistical significance was determined at P < 0.05. Anxiety and depression state data are expressed as percentages and P value was determined at P < 0.05 (Student’s t test for unpaired data).

## Results

### Patients

Characteristics of the study population are summarized in Table 1. No significant differences in terms of age and BMI were observed between RLP and control women. Only three of seventy couples recruited in this study obtained their pregnancies by assisted reproductive technologies (ART). In particular, one couple underwent two in vitro fertilizations (IVF) and one spontaneous pregnancy; the other two couples underwent an IVF and two spontaneous pregnancies. None of the couples enrolled in this study had conceived with donated oocytes. Because of the early gestational age of the spontaneous pregnancy losses, no information is available from any of the couples enrolled in the RPL group about the chromosomal results on the product of conception.

**Table 1:** Clinical characteristics and result of clinical and molecular tests administered to RPL and controls women

	CTR [n = 30]	RPL [n = 70]	P value
Age (years) ± SD	34.7 ± 6.7	37.3 ± 4.3	NS
BMI (Kg/m^2^)	21.9 ± 2.8	24.4 ± 3.4	NS
IVF (n)	0.0 (0.0%)	3.0 (4.3%)	NS
Intestinal permeability (%) ± SD	2.2 ± 0.3	6.4 ± 0.7	< 0.0001
Serum LPS levels (EU/ml) ± SD	0.3 ± 0.1	0.9 ± 0.1	< 0.05
Anxiety state (STAI-Y1) (%)	35.0	87.0	< 0.05
Anxiety trait (STAI-Y2) (%)	31.0	60.0	< 0.05
Depression state (Zung scale) (%)	24.0	48.0	< 0.05
Abnormal endometrial Nalp-3 expression (%)	0.3	85.7 [n = 35]	< 0.0001

Results are expressed as average ± standard deviation (SD) or percentage (%) of total

*CTR* controls, *n* number, *BMI* body mass index, *IVF* in vitro fertilization, *NS* not statistically significant, *RPL* recurrent pregnancy loss, *LPS* lipopolysaccharide

### RPL was associated with abnormal intestinal permeability

Intestinal permeability of enrolled patients was assessed by ^51^Cr-EDTA absorption test. We observed a higher prevalence of abnormal intestinal permeability in RPL patients compared to the control group (Table 1). At gastrointestinal evaluation, none of women with increased intestinal permeability referred clear symptoms of gastrointestinal diseases. None of the recruited women showed any abnormal levels of immunoglobulin (Ig) A or E, or any positivity for anti-endomysial, anti-gliadin, anti-transglutaminase antibodies, HLA DQ2-DQ8 haplotype, lactose/lactulose/sorbitol breath tests, fecal calprotectin assay, pANCA, cANCA, ASCA or ASMA antibodies.

### RPL women showed higher circulating levels of lipopolysaccharides

Assessment of serum LPS levels revealed significantly increased circulating levels of LPS in women with RPL compared to the controls (P < 0.05; Table 1). The cut off for defining abnormal LPS circulating levels was established to be > 2SD of the LPS average levels in a healthy population (0.4 EU/ml).

### Women with RPL showed a higher prevalence of depression and anxiety

Results of the psychological tests are reported in Table 1. Overall, RPL patients showed more symptoms of depression and anxiety than control healthy subjects.

Eighty-seven percent of the RPL women, compared with 35% of the control group, had STAI-Y1 (state anxiety) scores > 39 (95% mild and 5% moderate for the RPL group; 90% mild and 10% moderate for the control group).

Sixty percent of the RPL women, compared with 31% of the control group, had STAI-Y2 (trait anxiety) scores > 39 (79% mild and 21% moderate for the RPL group; 89% mild and 11% moderate for the control group).

Forty-eight percent of the RPL women, compared with 24% of the healthy control group, had a score > 50 on the Z-SDS test (40% mildly depressed and 8% moderately depressed; P < 0.05).

### Endometrial Nalp-3 inflammasome was over expressed and activated in RPL

**Fig. 1:**
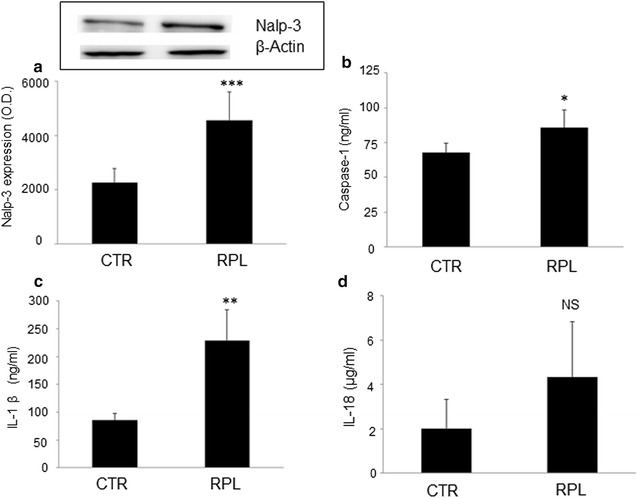
**a** Representative Western blot analysis of endometrial Nalp-3 inflammasome expression. Endometrial lysates from RPL (n = 10) showed significantly higher expression of Nalp-3 compared to controls (n = 10) (*O.D* optical density). Histograms show average ± SD of three independent experiments. **b**, **c** Endometrial Nalp-3 inflammasome activation assessed by ELISA. Significantly higher levels of endometrial caspase-1 (**b**) and Il-1β (**c**) were observed in RPL patients (n = 35) compared to controls (n = 20). Histograms show average ± SD of three independent experiments. CTR: controls. Statistical significance versus CTR: *P < 0.05; **P < 0.01; ***P < 0.0001. CTR: controls

Western Blot analysis showed a significant increase of Nalp-3 proteins expression in endometrial tissues of women with idiopathic RPL compared to the controls (P < 0.0001) (Table 1 and Fig. 1a). The cut off for defining Nalp-3 endometrial expression to be abnormal was > 2SD of endometrial Nalp-3 average expression of the control population (3000 O.D.).

We also examined the levels of caspase-1 in endometrial lysates obtained from hysteroscopic biopsies by ELISA. As shown in Fig. 1b, increased levels of caspase-1 were shown in the RPL women compared to the controls (P < 0.05). Finally, through an ELISA assay, we evaluated the levels of IL-1β and IL-18 in endometrial lysates obtained from the controls and RPL women. Significantly increased levels of pro-inflammatory cytokine IL-1β (Fig. 1c), but not of IL-18 (Fig. 1d), were found by ELISA in endometrial tissues of the RPL women compared to the controls (P < 0.001).

### Abnormal intestinal permeability and circulating LPS levels were associated with increased expression of endometrial Nalp-3 in women with RPL

When analyzing statistical correlation between abnormal intestinal permeability, LPS serum levels and endometrial Nalp-3 expression and activation in patients with RPL, we observed a clear association between abnormal intestinal permeability and endometrial Nalp-3, suggesting a possible cause-effect relation between passage of inflammatory stimuli through the gut barrier and the activation of endometrial innate immunity (Table 2). Neither intracellular activation of Nalp-3 cascade (caspase-1) nor secretion of the pro-inflammatory specific IL1β and IL-18 were observed to be statistically associated to abnormal intestinal permeability or LPS (data not shown). No statistical correlation was found between intestinal permeability abnormality and increased LPS serum levels in patients with RPL (Table 2). Significant correlation was observed between LPS serum levels and Nalp-3 endometrial expression in patients with RPL (P < 0.01) (Table 3).

**Table 2:** Statistical correlation between abnormal intestinal permeability and circulating levels of LPS, endometrial Nalp-3 inflammasome expression, anxiety and depression state in women with RPL

	Intestinal permeability		P value
	Normal	Abnormal	
LPS serum levels [n = 70]			
Normal	4 (5.7%)	10 (14.3%)	0.8
Abnormal	5 (7.1%)	51 (72.9%)	
Abnormal endometrial Nalp-3 expression [n = 35]			
Normal	3 (8.6%)	2 (5.7%)	< 0.01
Abnormal	4 (11.4%)	26 (74.3%)	
Anxiety state (STAI-Y1) [n = 70]			
Absent	1 (1.4%)	6 (8.6%)	0.9
Present	1 (1.4%)	62 (88.6%)	
Anxiety trait (STAI-Y2) [n = 70]			
Absent	1 (1.4%)	12 (17.2%)	0.3
Present	1 (1.4%)	56 (80.0%)	
Depression state (Zung scale) [n = 70]			
Absent	1 (1.4%)	22 (31.4%)	0.6
Present	1 (1.4%)	46 (65.8%)	

Results are expressed as percentage (%) of total. *LPS* lipopolysaccharide. P < 0.05. *n* number

**Table 3:** Statistical correlation between LPS serum levels and endometrial Nalp-3 inflammasome expression, anxiety and depression state in women with RPL

	LPS levels		P value
	Normal	Abnormal	
Nalp-3 expression [n = 35]			
Normal	7 (20%)	1 (2.9%)	< 0.01
Abnormal	8 (22.9%)	19 (54.2%)	
Anxiety state (STAI-Y1) [n = 70]			
Absent	1 (1.4%)	5 (7.1%)	0.8
Present	18 (25.8%)	46 (65.7%)	
Anxiety trait (STAI-Y2) [n = 70]			
Absent	1 (1.4%)	15 (21.4%)	< 0.05
Present	15 (21.4%)	39 (55.8%)	
Depression state (Zung scale) [n = 70]			
Absent	10 (14.3%)	10 (14.3%)	0.6
Present	10 (14.3%)	40 (57.1%)	

Results are expressed as percentage (%) of total. *LPS* lipopolysaccharide. P < 0.05. *n* number

### Abnormal intestinal permeability and circulating LPS levels are not associated to anxiety and depression state in women with RPL

Abnormal intestinal permeability was not associated to anxiety state or trait, or depression (Table 2). RPL women with higher circulating levels of LPS did not show significant higher prevalence of anxiety and depression state compared to once with normal levels of serum LPS (Table 3). A significant association was found only between abnormal serum levels of LPS and anxiety trait (Table 3). As a whole, abnormal intestinal permeability and serum LPS levels do not seem to be related to psychological disorder in a strong cause-effect relationship.

## Discussion

The main finding of the present study is the higher prevalence of abnormal intestinal permeability in patients with idiopathic RPL. In the same population, we detected higher plasma levels of LPS, an immunogenic parietal fragment from Gram-negative bacteria. Furthermore, consistently with a previous study [12], we confirmed an increased expression and activation of Nalp-3 inflammasome proteins in the human endometrium of women with RPL, as well as an increased caspase-1 activation and secretion of the pro-inflammatory cytokine IL-1β. Interestingly, for the first time, we could link the endometrial Nalp-3 inflammasome over expression and activation with leaky gut. Based on our data, we can speculate that in RPL women bacterial components might enter systemic circulation through a damaged intestinal barrier and induce inflammatory cytokines production in endometrial tissues, via inflammasome Nalp-3 activation. The lack of significant correlation between increased intestinal permeability and LPS might be explained: (a) because of the small number of patients recruited; (b) for the heterogeneity of gut microbiota composition in our population; (c) because not only bacteria-derived molecules but also other molecules coming from the external environment (food, chemicals, drugs, etc.) might cross gut barrier and stimulate Nalp-3 inflammasome. Thus, we hypothesize that leaky gut, occurring for reasons that we cannot explain yet, allows the passage of antigens through the intestinal barrier, that might elicit innate immunity in endometrial tissue. Hence, when there is an increased intestinal permeability and/or when higher circulating levels of LPS are detected in RPL subjects, there might be a higher risk of endometrial inflammation and reproductive disorders.

The occurrence of cross-talk between the gut and the reproductive system may be an intriguing hypothesis that could suggest a possible new approach to a specific group of patients with idiopathic RPL. Evidence supporting a role for the intestinal-endometrial axis in the pathogenesis of early pregnancy complications has being slowly accumulating over the most recent years [20, 21]. The most studied pathogenic model is celiac disease (CD). CD has been related to augmented intestinal permeability due to the ability of gliadin to disrupt intestinal epithelial tight junction proteins including zonulin-1, claudin-1 and occludin, all polyamines with a pivotal role in the control of intestinal barrier function, inducing the leaky gut syndrome [22, 23]. Several studies reported an increased risk of reproductive failures including recurrent miscarriage in women with CD. In particular, according to a previous meta-analysis, women experiencing RPL have an Odds Ratio (OR) for CD of 5.82 (95% CI 2.30–14.74) and the risk of miscarriage in women with CD is significantly higher with an RR of 1.39 (95% CI 1.15–1.67) [20]. As a whole, we can hypothesize that recovering intestinal barrier might reduce tissue inflammation and take the endometrium back to a favorable environment for implantation and pregnancy development.

Finally, the role of anxiety and depression in RPL patients has yet to be clarified. We did not find a strong correlation among depression/anxiety state and intestinal permeability or abnormal LPS levels but only an association between abnormal LPS circulating levels and anxiety trait. However, in our population of RPL, we found a higher prevalence of self-reported stress and moderate/severe depression. Previous studies on depression and RPL have been few, using a variety of scales and lacking an appropriate comparison group [24–27]. The reported prevalence of depression varies considerably across studies, ranging from 15 to 33% [24–27]. Psychological stress and RPL have been not extensively studied, but one case–control study described a significantly higher psychological stress total score among 45 patients with RPL, compared with 40 controls [10]. Interestingly, it has been demonstrated that the gut-brain axis involves bidirectional communication between the central nervous system and the gastrointestinal tract via neurocrine and endocrine signaling pathways [28]. Indeed, physical and psychological stressors can alter the gut microbiota’s composition and metabolic activities, and signals produced by the gut microbiota can in turn affect the brain and emotional responses [29]. Moreover, depression can promote intestinal permeability leading to leaky gut. Interestingly, depressed patients have been found to have higher antibodies against gut bacteria than healthy subjects [30]. In another study, patients with major depression showed higher circulating levels of 16S rDNA, a marker of bacterial translocation, compared with healthy subjects, and the magnitude was correlated with depressive symptom severity [31]. As a whole, it is widely accepted that increased bacterial translocation could play a role in the inflammatory pathophysiology of depression by inducing acute inflammatory responses and contributing to subchronic inflammation [32]. To support these evidences, LPS has been shown to elicit depressive and anxious behaviors in rats [33].

## Conclusions

Leaky gut, depressive symptoms and RPL are intertwined conditions but it is difficult to establish which cause-effect relationship might occur. This is the first study detecting abnormal intestinal permeability in women with RPL. According to our hypothesis, in this population an abnormal intestinal permeability, worsened or caused ex novo by a depressive or anxious state, might allow passage of immune triggers from external environment, not only of bacterial derivation, that might elicit innate immune response, leading to endometrial inflammation and, thus, potentially to miscarriage.

More studies are needed to confirm our hypothesis and define the pathogenic mechanisms of inflammation-induced miscarriage. Furthermore, the beneficial effect of recovering intestinal barrier function in RPL women, as well as psychological health, on subsequent obstetric outcomes should be evaluated.

## Abbreviations

^51^Cr-EDTA: ^51^Cr-ethylene-diamine-tetraacetic acid
ANOVA: one-way analysis of variance
ASC: apoptosis-associated speck-like protein containing a CARD
cANCA: cytoplasmic anti-neutrophil cytoplasmic antibodies
ELISA: enzyme linked immunosorbent assay
HLA: human leukocyte antigen
IL: interleukin
LPS: lipopolysaccharide
pANCA: perinuclear anti-neutrophil cytoplasmic antibodies
RPL: recurrent pregnancy loss
STAI-Y test: State-Trait Anxiety Inventory Y test
Z-SDS: Zung Self-Rating Depression Scale
