# Correction to: Recurrent pregnancy loss is associated to leaky gut: a novel pathogenic model of endometrium inflammation?

**DOI:** 10.1186/s12967-019-1823-5

**Published:** 2019-03-15

**Authors:** C. Tersigni, S. D’Ippolito, F. Di Nicuolo, R. Marana, V. Valenza, V. Masciullo, F. Scaldaferri, F. Malatacca, C. de Waure, A. Gasbarrini, G. Scambia, N. Di Simone

**Affiliations:** 1 U.O.C. di Ostetricia e Patologia Ostetrica, Dipartimento di Scienze della Salute della Donna e del Bambino, Fondazione Policlinico Universitario A. Gemelli IRCCS, Rome, Italy; 2 Istituto di Clinica Ostetrica e Ginecologica, Università Cattolica del Sacro Cuore, Rome, Italy; 3 Department of Woman and Child Health, A. Gemelli Hospital, Università Cattolica Del Sacro Cuore of Rome, 00168 Rome, Italy; 4 International Scientific Institute Paolo VI, ISI, A. Gemelli Hospital, Università Cattolica Del Sacro Cuore, 00168 Rome, Italy; 5 Department of Nuclear Medicine, A. Gemelli Hospital, Università Cattolica Del Sacro Cuore, A. Gemelli Hospital, 00168 Rome, Italy; 6 U.O.C. di Ginecologia Oncologica, Dipartimento di Scienze della Salute della Donna e del Bambino, Fondazione Policlinico Universitario A. Gemelli IRCCS, Rome, Italy; 7 Department of Internal Medicine, A. Gemelli Hospital, Università Cattolica Del Sacro Cuore, 00168 Rome, Italy; 8 Institute of Public Health, A. Gemelli Hospital, Università Cattolica Del Sacro Cuore, 00168 Rome, Italy

## Correction to: J Transl Med (2018) 16:102 10.1186/s12967-018-1482-y

Following publication of the original article [1], the authors reported updated affiliations for five of the authors. The updated affiliations are shown below and reflected in the affiliation list of this Correction.

The corrected affiliations for C. Tersigni, S. D’Ippolito and N. Di Simone are:

- Fondazione Policlinico Universitario A. Gemelli IRCCS, U.O.C. di Ostetricia e Patologia Ostetrica, Dipartimento di Scienze della Salute della Donna e del Bambino, Rome, Italy
- Università Cattolica del Sacro Cuore, Istituto di Clinica Ostetrica e Ginecologica, Rome, Italy

The corrected affiliations V. Masciullo and G. Scambia are:

- Università Cattolica del Sacro Cuore, Istituto di Clinica Ostetrica e Ginecologica, Rome, Italy
- Fondazione Policlinico Universitario A. Gemelli IRCCS, U.O.C. di Ginecologia Oncologica, Dipartimento di Scienze della Salute della Donna e del Bambino, Rome, Italy
